# Supplementary material for: Unlocking River Biofilm Microbial Diversity: A Comparative Analysis of Sequencing Technologies
Source: Mol Ecol Resour. 2025 Nov 6;26(1):e70075. doi: 10.1111/1755-0998.70075 (PMC12627904; doi:10.1111/1755-0998.70075)
Supplement: Supplementary file 1 — Data S1: men70075‐sup‐0001‐DataS1.zip. [file MEN-26-e70075-s001.zip › men70075-sup-0001-Supinfo01.docx]

## Supplementary Material

Supplementary Table 1: Primers used in this study.

| Sequencing Method | Forward primer (5’-3’) | Reverse primer (5’-3’) | Reference |
| --- | --- | --- | --- |
| Illumina 1st round | 16S rRNA 515f:  GTGYCAGCMGCCGCGGTAA | 16S rRNA 806r: GGACTACNVGGGTWTCTAAT | Walters et al. 2016 |
| Illumina 1st round adaptor | Forward adaptor: TCGTCGGCAGCGTCAGATGTGTATAAGAGAC | Reverse adaptor: GTCTCGTGGGCTCGGAGATGTGTATAAGAGACAG |  |
| Pacific Biosciences | Kinnex 16S F:  AGRGTTYGATYMTGGCTCAG | Kinnex 16S R:  RGYTACCTTGTTACGACTT | Kinnex V1-V9 |

Supplementary Table 2: PCR conditions.

| Sequencing Method | Stage | Temperature °C | Time | Number of cycles |
| --- | --- | --- | --- | --- |
| Illumina 1st round | Initial denaturation  Denaturation  Annealing  Extension  Final extension | 95  95  50  72  72 | 2 min  15 sec  30 sec  30 sec  10 min | 30 |
| Illumina  Indexing | Initial denaturation  Denaturation  Annealing  Extension  Final extension | 95  95  50  72  72 | 2 min  15 sec  30 sec  30 sec  10 min | 8 |
| Pacific Biosciences | Initial denaturation  Denaturation  Annealing  Extension  Final extension | 95  98  57  72  72 | 3 min  20 sec  30 sec  75 sec  5 min | 20 |

Supplementary Table 4: The number of reads in each sample pre-filtering, pre-rarefaction and post-rarefaction. Samples were rarefied to 3000 reads.

| Sample | Sequencing  type | Pre-DaDa2 | Pre-rarefaction reads | Post-rarefaction reads |
| --- | --- | --- | --- | --- |
| A30l | Long | 7803 | 7514 | 3000 |
| A62l | Long | 6266 | 3252 | 3000 |
| B17l | Long | 3773 | 3386 | 3000 |
| B23l | Long | 5016 | 4782 | 3000 |
| B39l | Long | 6505 | 4563 | 3000 |
| B57l | Long | 6409 | 3952 | 3000 |
| C39l | Long | 6114 | 5466 | 3000 |
| C49l | Long | 5435 | 4204 | 3000 |
| E31l | Long | 4806 | 3654 | 3000 |
| E43l | Long | 4878 | 4791 | 3000 |
| F2l | Long | 4360 | 3939 | 3000 |
| F34l | Long | 5792 | 5698 | 3000 |
| F35l | Long | 8686 | 7707 | 3000 |
| F65l | Long | 5937 | 4265 | 3000 |
| G54l | Long | 5052 | 4097 | 3000 |
| H61l | Long | 5602 | 5270 | 3000 |
| I1l | Long | 4836 | 3954 | 3000 |
| I55l | Long | 4471 | 4449 | 3000 |
| J27l | Long | 3533 | 3222 | 3000 |
| J6l | Long | 3893 | 3863 | 3000 |
| L13l | Long | 8046 | 5634 | 3000 |
| L29l | Long | 4932 | 4850 | 3000 |
| L35l | Long | 6199 | 6025 | 3000 |
| L43l | Long | 4953 | 4117 | 3000 |
| L44l | Long | 4923 | 4564 | 3000 |
| L48l | Long | 6686 | 4945 | 3000 |
| L53l | Long | 6859 | 6697 | 3000 |
| M28l | Long | 5654 | 3095 | 3000 |
| M62l | Long | 3697 | 3684 | 3000 |
| N41l | Long | 4673 | 4645 | 3000 |
| N45l | Long | 5788 | 4559 | 3000 |
| O53l | Long | 5125 | 4399 | 3000 |
| P22l | Long | 4343 | 4090 | 3000 |
| P50l | Long | 5924 | 3532 | 3000 |
| S28l | Long | 5027 | 4729 | 3000 |
| T62l | Long | 5190 | 4896 | 3000 |
| U13l | Long | 7875 | 4429 | 3000 |
| P3l | Long | 2873 | 2642 | NA |
| D16l | Long | 6602 | 2884 | NA |
| J59l | Long | 4936 | 2900 | NA |
| A60l | Long | 5443 | 2980 | NA |
| B43l | Long | 1104 | 1024 | NA |
| A30s | Short | 8732 | 8716 | 3000 |
| A62s | Short | 7387 | 7300 | 3000 |
| B17s | Short | 8840 | 8831 | 3000 |
| B23s | Short | 8695 | 8674 | 3000 |
| B39s | Short | 5045 | 5029 | 3000 |
| B57s | Short | 6658 | 6593 | 3000 |
| C39s | Short | 8453 | 8401 | 3000 |
| C49s | Short | 7193 | 7177 | 3000 |
| E31s | Short | 7106 | 7065 | 3000 |
| E43s | Short | 9172 | 9165 | 3000 |
| F2s | Short | 6835 | 6758 | 3000 |
| F34s | Short | 9071 | 9066 | 3000 |
| F35s | Short | 8404 | 8352 | 3000 |
| F65s | Short | 8445 | 8377 | 3000 |
| G54s | Short | 7684 | 7501 | 3000 |
| H61s | Short | 8459 | 8364 | 3000 |
| I1s | Short | 7715 | 7672 | 3000 |
| I55s | Short | 9042 | 9038 | 3000 |
| J27s | Short | 8784 | 8769 | 3000 |
| J6s | Short | 9113 | 9110 | 3000 |
| L13s | Short | 7737 | 7685 | 3000 |
| L29s | Short | 9124 | 9120 | 3000 |
| L35s | Short | 8543 | 8532 | 3000 |
| L43s | Short | 8715 | 8701 | 3000 |
| L44s | Short | 8243 | 8236 | 3000 |
| L48s | Short | 7840 | 7799 | 3000 |
| L53s | Short | 8408 | 8402 | 3000 |
| M28s | Short | 6421 | 4651 | 3000 |
| M62s | Short | 8636 | 8635 | 3000 |
| N41s | Short | 8215 | 8215 | 3000 |
| N45s | Short | 7858 | 7820 | 3000 |
| O53s | Short | 7896 | 7829 | 3000 |
| P22s | Short | 8285 | 8145 | 3000 |
| P50s | Short | 7273 | 7148 | 3000 |
| S28s | Short | 8400 | 8363 | 3000 |
| T62s | Short | 9209 | 9204 | 3000 |
| U13s | Short | 6988 | 6727 | 3000 |
| P3s | Short | 8312 | 8301 | NA |
| D16s | Short | 4970 | 4941 | NA |
| J59s | Short | 6522 | 6489 | NA |
| A60s | Short | 6045 | 6007 | NA |
| B43s | Short | 8397 | 8358 | NA |


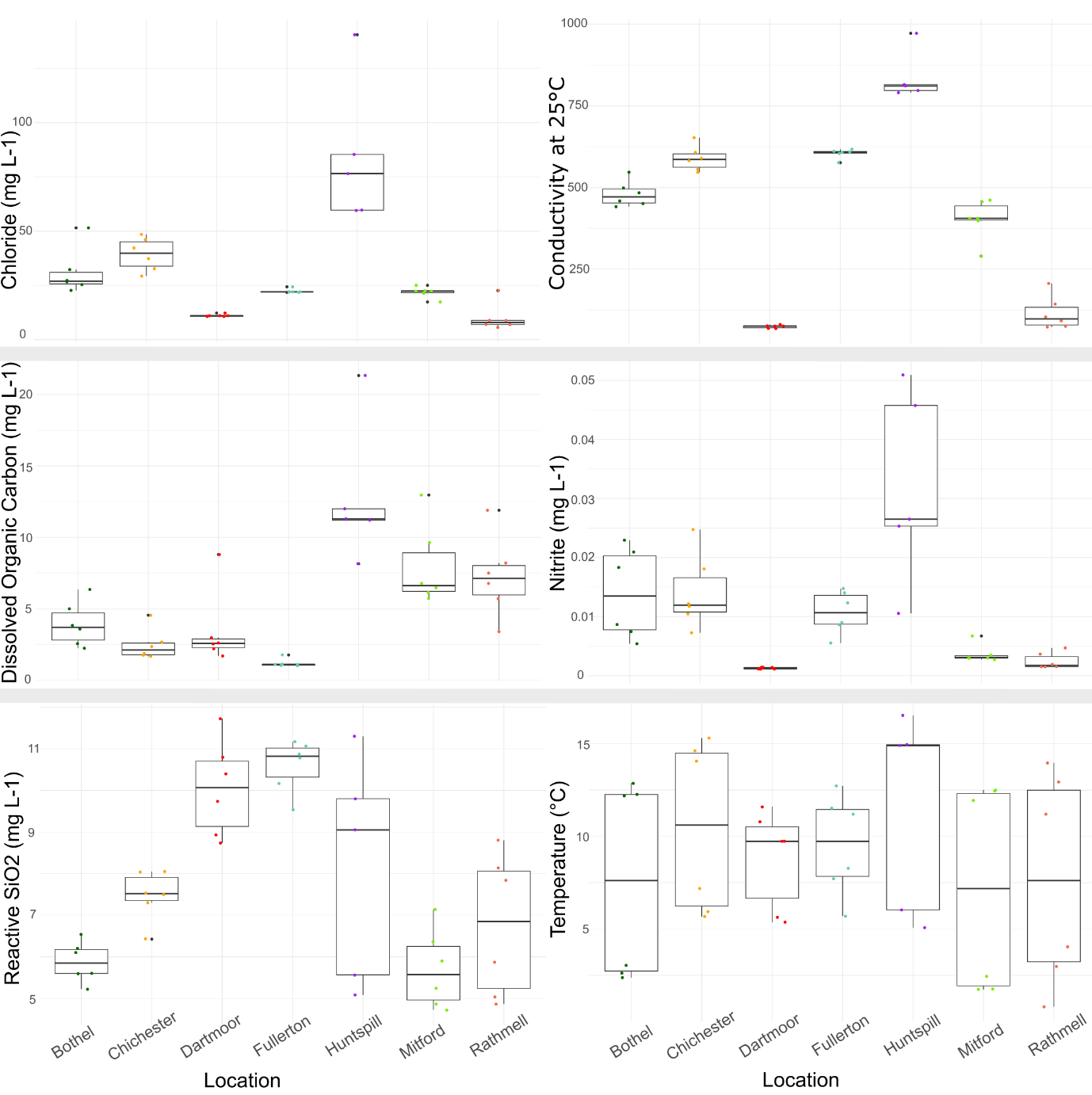


Supp Figure 1: chemistry data for the seven sampling sites; Chloride, Conductivity, Dissolved Organic Carbon, Nitrite, Reactive SiO2, and Temperature.

Supp Figure 2: Observed, Chao1 and Shannon Alpha diversity index for short-read and long-read sequencing. Shown as box plots and violin plots.


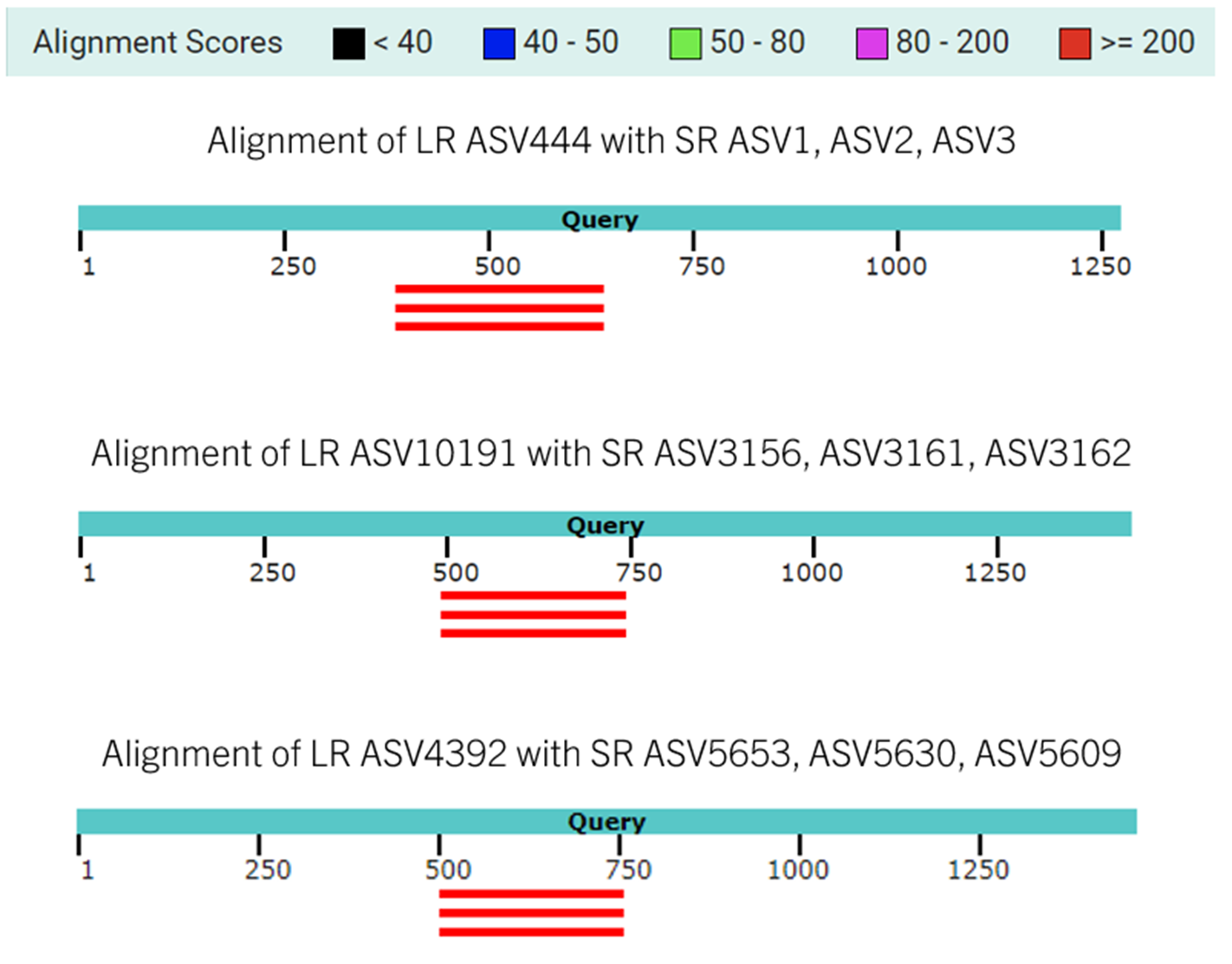


Supp Figure 3: Visualisation of BLAST alignments between long-read (LR) and short-read (SR) ASVs. The blue bar represents the LR ASV query sequence, while the red bars indicate the aligned SR ASVs that match the LR sequence. Alignment scores are colour-coded along the top bar, with red indicating a stronger alignment.


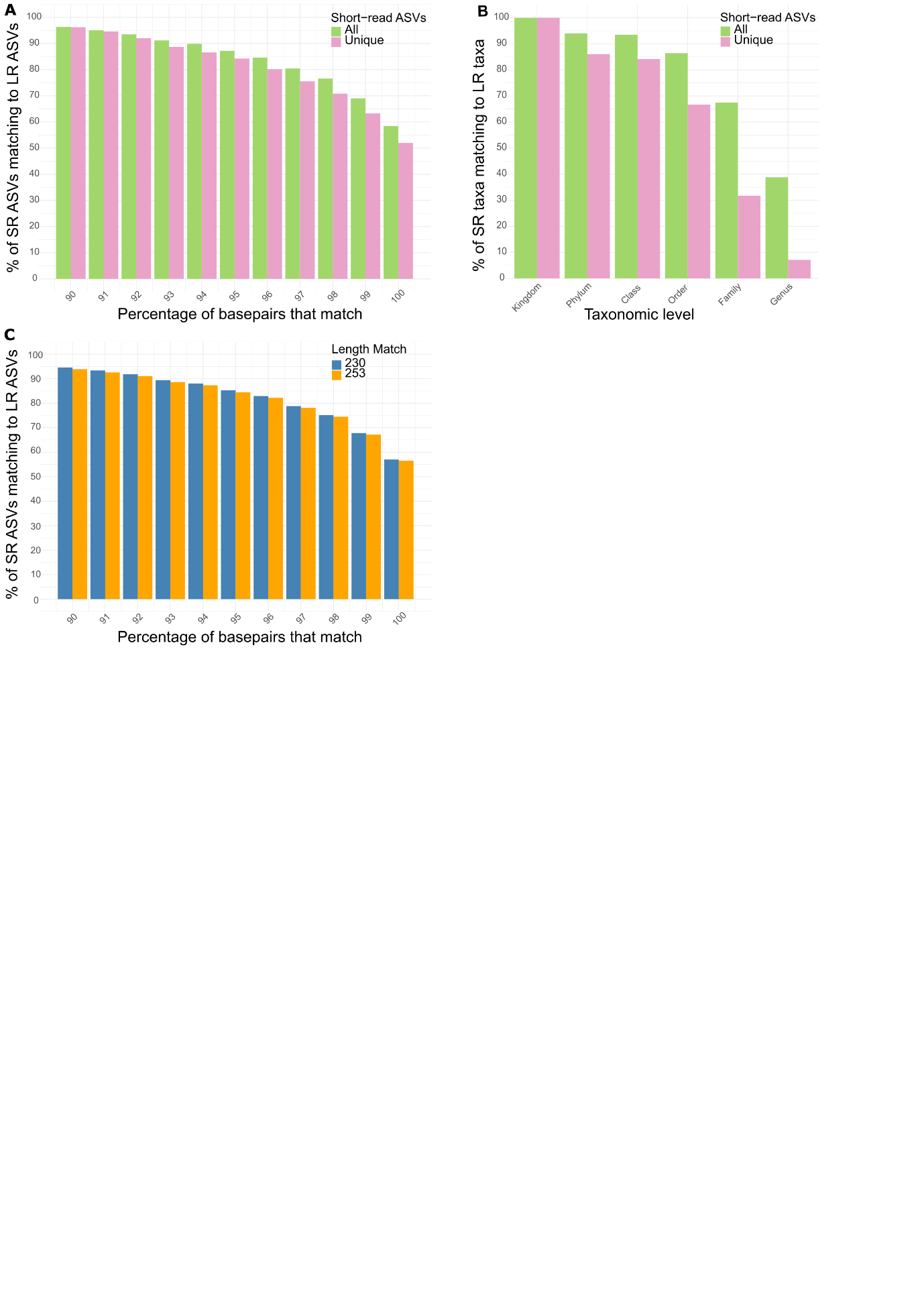


Supp Figure 4: Comparison of short-read (SR) ASVs mapped to long-read (LR) ASVs. (A) Percentage of short-read ASVs matching long-read ASVs across different sequence identity thresholds, with "All” SR ASVs in green and "Unique" SR ASVs in pink. (B) Taxonomic overlap of short-read ASVs mapped to long-read ASVs at different classification levels. (C) Effect of short-read ASV length (230 bp vs. 253 bp) on the matching probability to long-read ASVs.


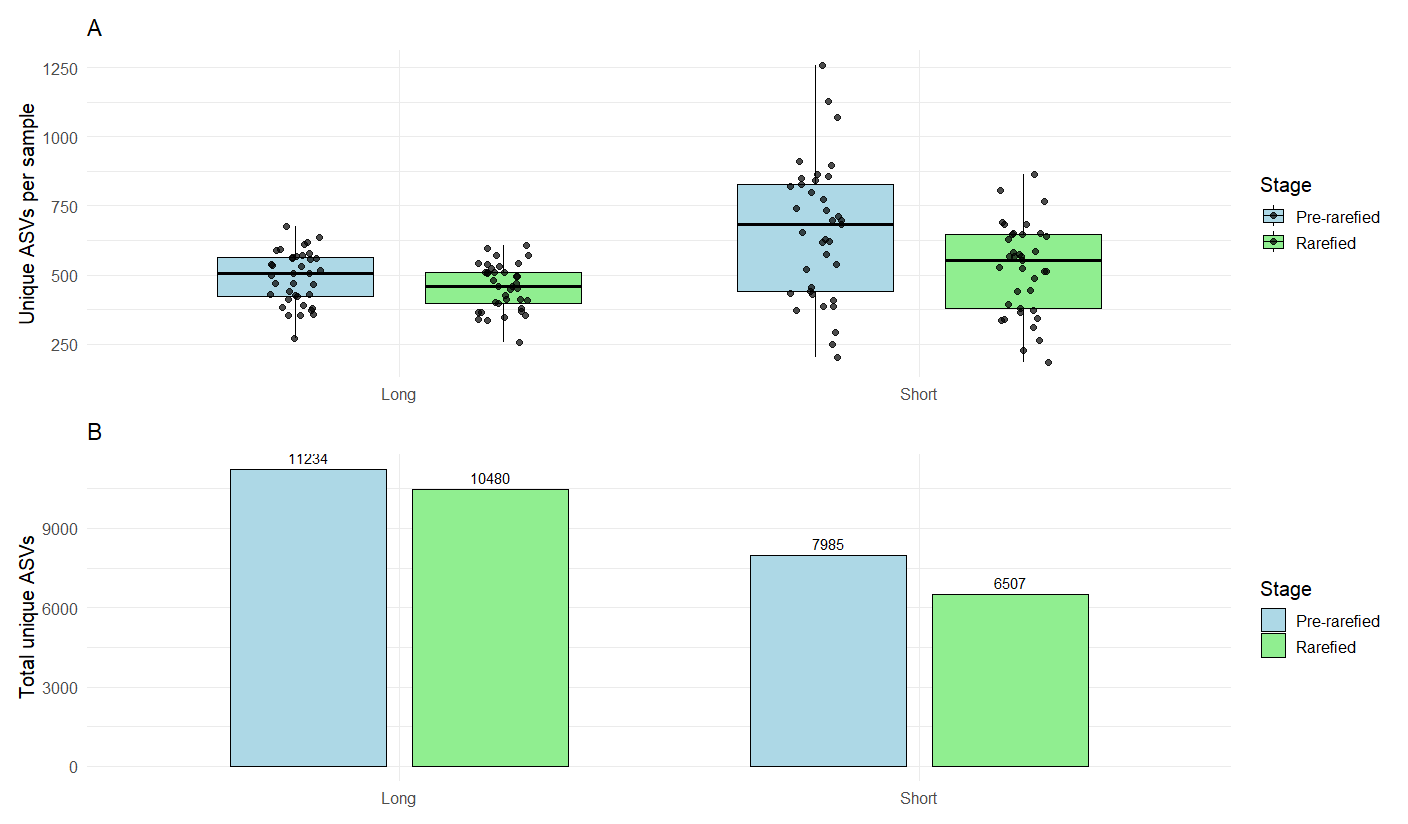


Supp Figure 5: The number of unique ASVs pre and post rarefaction for short-read and long-read sequencing. (A) Number of unique ASVs at the per sample level and (B) whole dataset level.


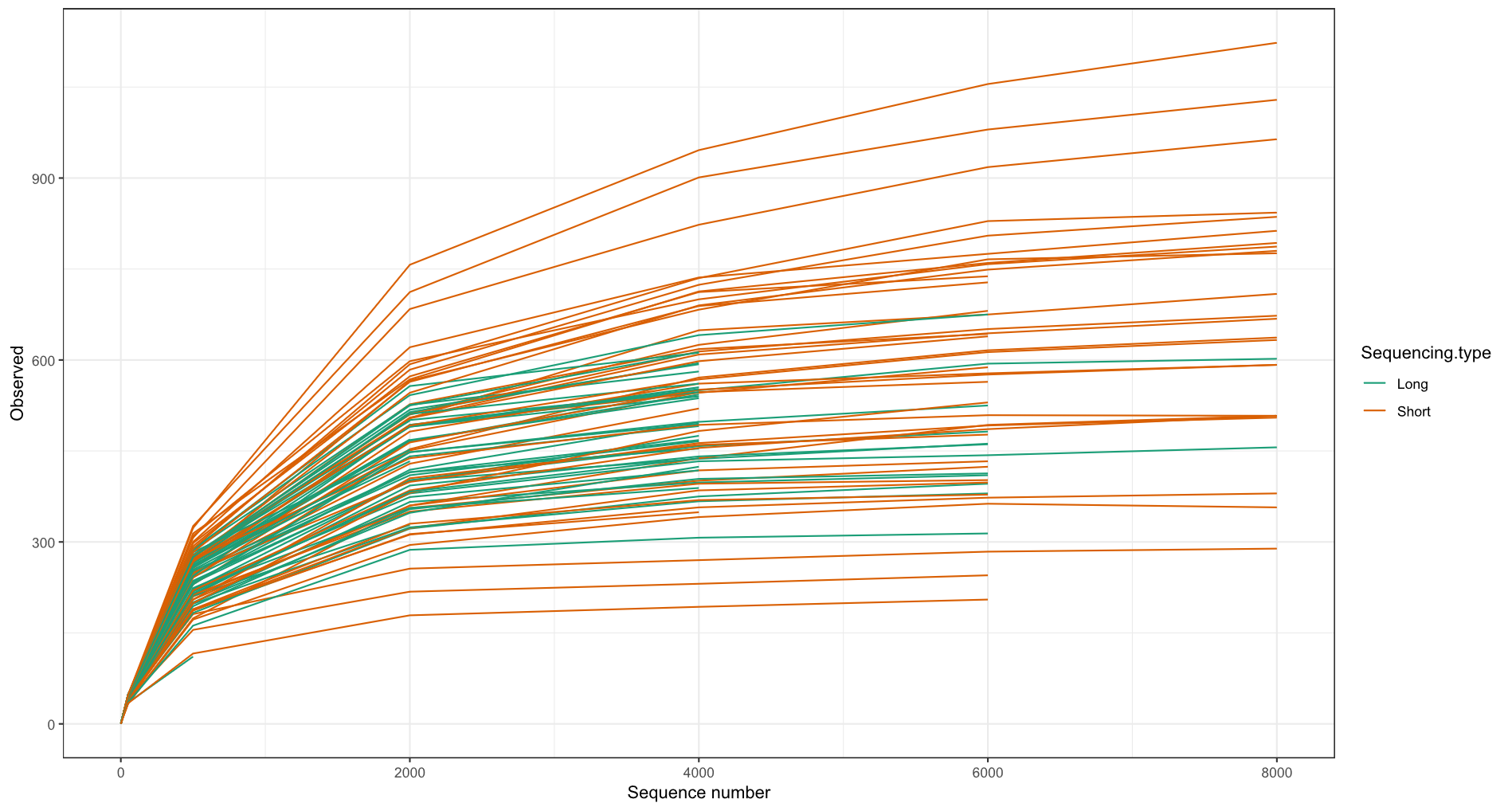


Supp Figure 6: Rarefaction curve for both long-read and short-read samples.


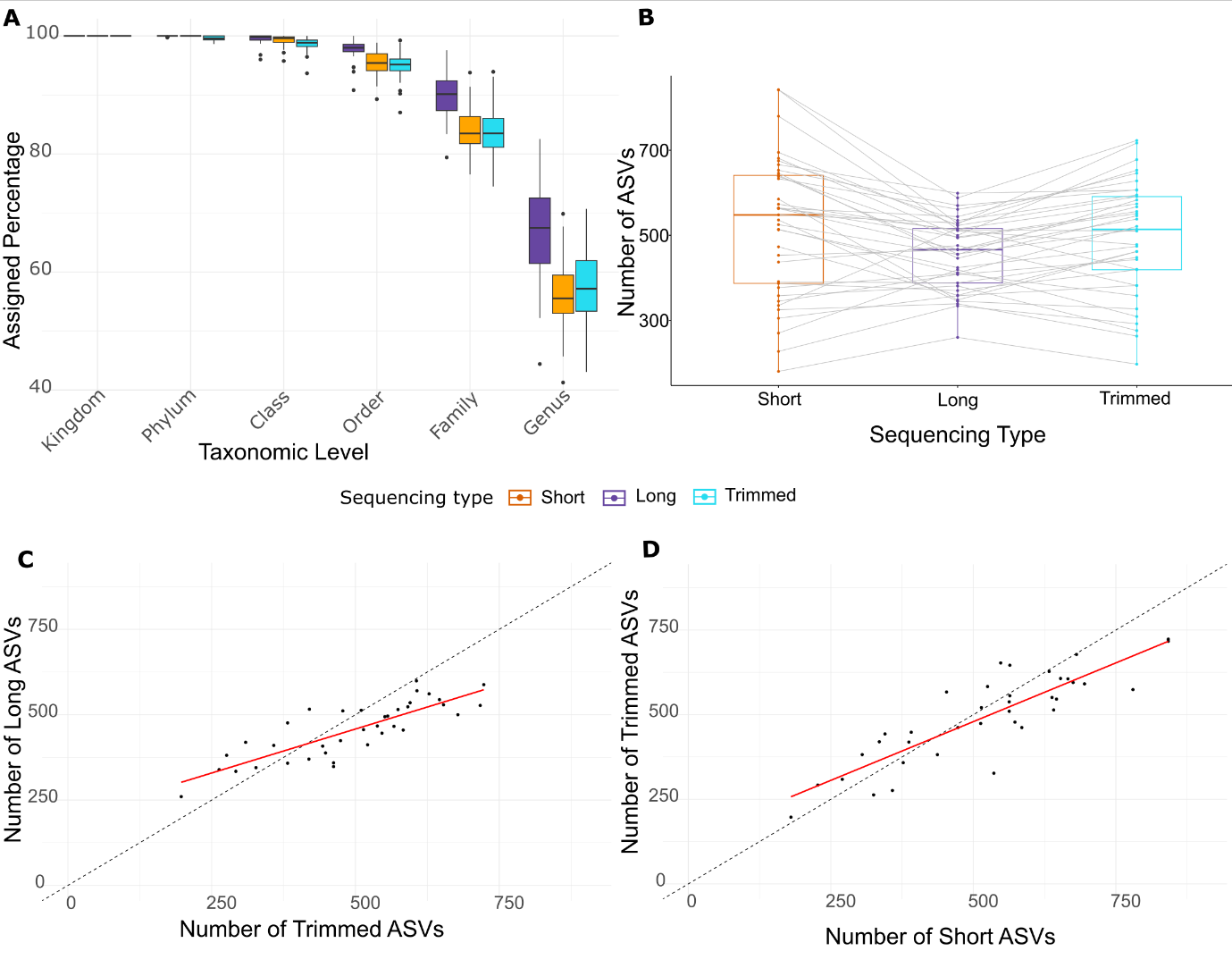


Supp Figure 7: Comparison of taxonomic assignment and ASV detection between long-read (purple), short-read (orange) and trimmed long-read (blue) sequences. (A) Percentage of assigned taxa at different taxonomic levels for long-, trimmed long- and short-read sequencing. (B) Paired comparison of ASV counts per sample. Relationship between ASV counts detected by long-read and trimmed long-read (C), and short-read and trimmed long-read (D) sequences, where the dashed black line represents a 1:1 ratio (perfect agreement), and the red line represents the fitted linear model (LLM).


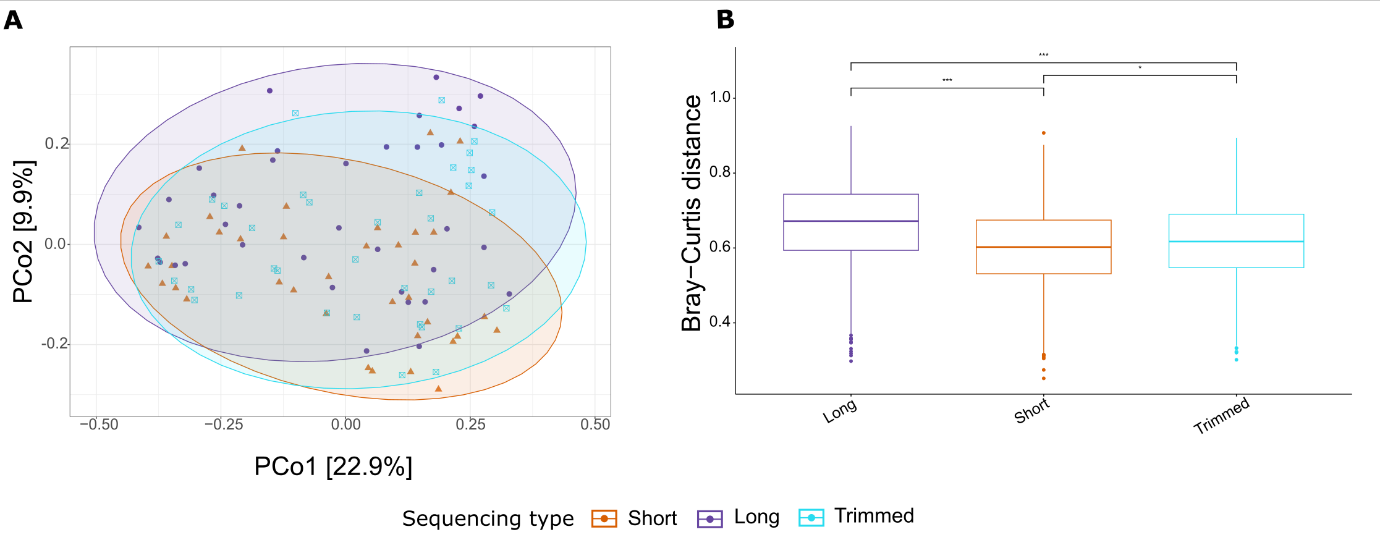


Supp Figure 8: PCoA of paired samples for short- (orange), long-read (purple) and trimmed long-read (blue) sequences (A). Bray-Curtis distance plot for the same samples (B)


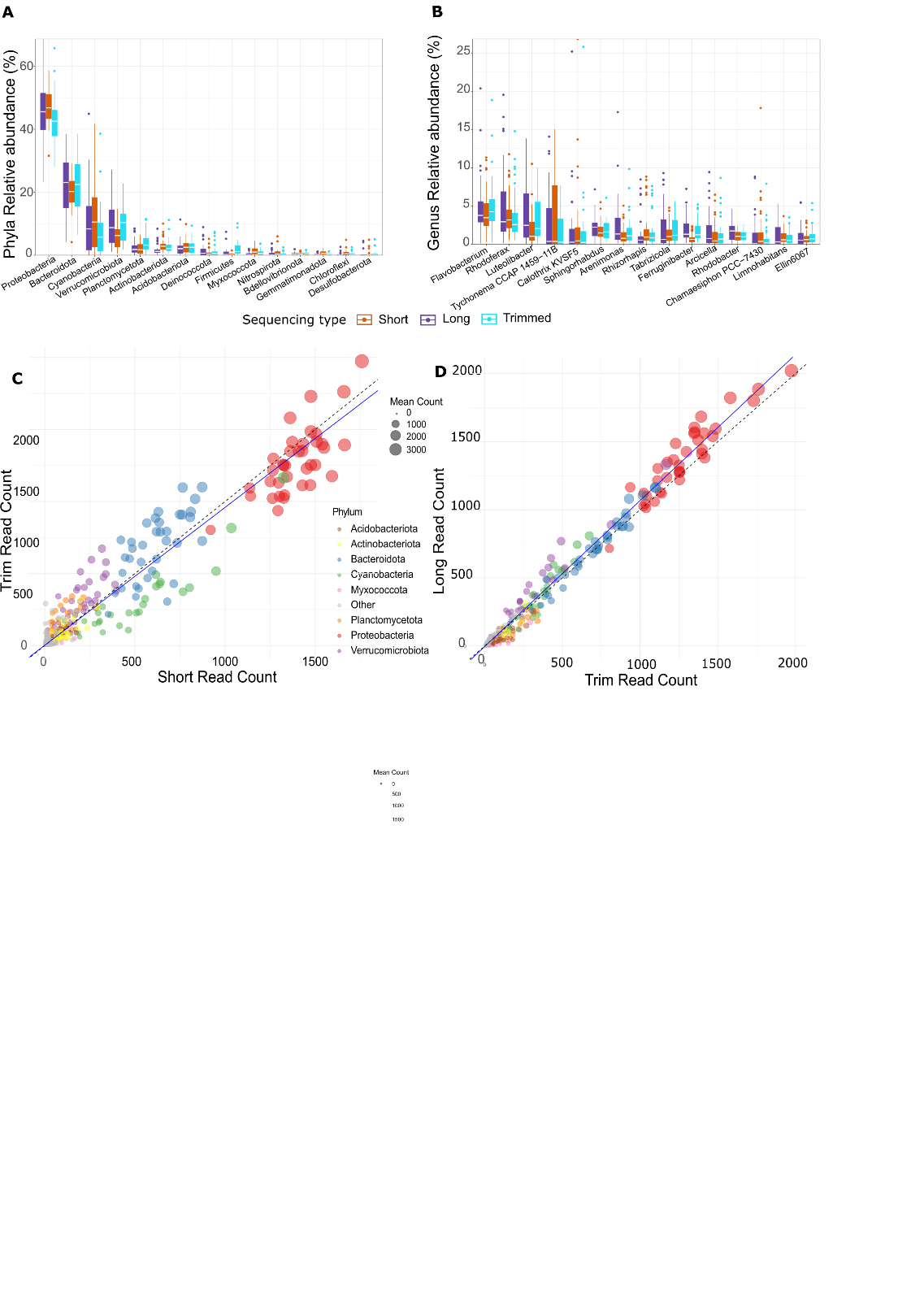


Supp Figure 9: Comparison of bacterial taxon composition between short- (orange), long-read (purple) and trimmed long-read (blue) sequences. Comparison of the top 15 abundant taxa at the phylum (A) and genus (B) levels for short-, long-, and trimmed-reads. Scatter plot showing the relationship between short-read and trimmed long read (C) and long-read and trimmed long-read (D)abundance for each phylum across all the samples. The top eight phyla are colour-coded, and the circle size is proportional to the mean number of reads per phylum in each paired sample. The dashed black line represents the line of perfect fit (1:1), and the blue line depicts the Deming regression line, with a slope of 0.957 (C) and 1.072 (D).
